# Tickborne Encephalitis, Southwestern France

**DOI:** 10.3201/eid1307.070041

**Published:** 2007-07

**Authors:** Bruno Herpe, Isabelle Schuffenecker, Jérome Pillot, Denis Malvy, Benjamin Clouzeau, Nam Bui, Frederic Vargas, Didier Gruson, Hervé Zeller, Marie E. Lafon, Hervé Fleury, Gilles Hilbert

**Affiliations:** * University Hospital of Bordeaux, Bordeaux, France; † Institut Pasteur, Lyon, France

**Keywords:** Tickborne encephalitis, arbovirosis, southwestern France, dispatch

## Abstract

We report an autochthonous human case of tickborne encephalitis (TBE) in the Bordeaux area, southwestern France. The patient was a farmer who had severe encephalomyelitis. ELISA and neutralization assay of serum and cerebrospinal fluid established the diagnosis. This potential new endemic focus for TBE virus should be further investigated.

Tickborne encephalitis (TBE) is the most important human arboviral infection of the central nervous system in Europe and Russia (*1*,*2*). The disease is endemic to areas where transmission vectors (*Ixodes ricinus*, *I. persulcatus*) are distributed. During the past 10 years, the incidence of the disease has increased, particularly in Lithuania, Germany, Switzerland, and Poland, and human cases have been reported from new areas ( *3*–*5*; see also www.tbe-info.com/tbe.aspx). Tickborne encephalitis viruses (TBEV), such as dengue, yellow fever, West Nile, and Japanese encephalitis viruses, belong to the family *Flaviviridae*. Among the TBEV, 3 genotypes have been described (*6*): the European subtype transmitted by *I. ricinus* and the Siberian and Far Eastern subtypes transmitted by *I. persulcatus*. Large mammals, such as goats, sheep, and cattle, are important blood-feeding hosts for adult ixodid ticks. Because the virus is excreted in milk, small outbreaks may result from consumption of raw milk from sheep or goats. To our knowledge, only viruses of the western genotype have been isolated in Western Europe. In central and Western Europe, cases occur between April and November, and peak in June–July and September–October in relation to tick activity (*1*,*2*). The progression of incidence follows the development of tourism, trekking, and camping/hiking in virus-endemic countries (*3*). The European subtype virus usually produces a biphasic illness. The incubation period lasts an average of 7–14 days (range 2–28 days). Primary infection is generally associated with a flulike syndrome, but infection may be asymptomatic (40% of cases). In 5%–30% of clinical cases, a second neurologic phase may occur with aseptic meningitis (50% of the cases), meningoencephalitis (40%), or meningoencephalomyelitis (10%) (*1*,*2*,*7*,*8*). The Far Eastern subtype TBEV infections are considered to be associated with more severe cases (mortality rate 10%–20%) and more frequent neurologic sequelae (5%–30%) (*1*). Specific diagnosis depends essentially on the detection of immunoglobulin M (IgM) antibodies in serum or cerebrospinal fluid (CSF) by ELISA (*9*).

In France, 5–10 cases of TBE are reported each year. Cases have been mainly reported since 1968 from Alsace-Lorraine in northeastern France (*2*). We report here the first, to our knowledge, autochthonous case from the Aquitaine region in southwestern France.

## The Case

On May 28, 2006, a 70-year-old farmer, living in the area of Bordeaux, southwestern France, was admitted, febrile and comatose, to the intensive care unit of the “Pellegrin hospital” in Bordeaux. He had no relevant medical history and had not traveled abroad during the previous year. The fever had begun 36 hours earlier, accompanied by headache, nausea, and vomiting. An attached tick had been removed from patient’s thigh during the previous week. He had no history of unpasteurized milk consumption. Physical examination found a temperature of 38.5°C and tachycardia. The patient had an altered consciousness with a Glasgow Coma Score of 7. He exhibited normal osteotendinous and cranial nerve reflexes, had no Babinski sign but had notable nuchal rigidity. Assisted ventilation was needed. A blood sample was collected at admission and showed the following: leukocyte count 10,300/mm^3^ (neutrophils 7,180/mm^3^, lymphocytes 1,140/mm^3^); hemoglobin 14.2 g/L, platelet count 209,000/mm^3^, creatinine 158 µmol/L, prothrombin rate 94%. The cerebrospinal fluid (CSF) findings were consistent with viral meningoencephalitis: leukocyte count 620 cells/mm^3^, 45% lymphocytes, protein 1.25 g/L, glucose 0.6 g/L. The patient was treated with 12 g/day intravenous amoxicillin and acyclovir. On day 4, the Glasgow Coma Score was 15, but the patient exhibited hypotonic tetraplegia with no osteotendinous reflexes. Magnetic resonance imaging showed cervicoarthrosis myelopathy but no sign of myelitis. An electromyograph confirmed the diagnosis of peripheral polyneuropathy. Ten days later, rapid motor improvement of lower extremities was observed, and the patient was transferred on day 15 to a rehabilitation center. An incapacitating brachial diplegia with amyotrophy persisted after 6 months.

For diagnostic purpose, paired serum samples were analyzed. No IgM or IgG antibodies were detected against *Borrelia* spp*.*, *Leptospira* spp., *Mycoplasma* spp*.*, *Rickettsia* spp*.*, *Brucella* spp*.*, *Treponema pallidum,* HIV, hepatitis B virus, and hepatitis C virus. Results of PCR assays of CSF were negative for *Borrelia* spp*.*, Enterovirus, Adenovirus, varicella-zoster virus, herpes simplex virus, Epstein-Barr virus, and cytomegalovirus. IgM capture and IgG indirect in-house assays with native antigen (from Hypr 1953 TBEV strain, kindly provided by F. Heinz) were performed to detect IgM and IgG TBEV antibodies. The cut-offs were as follows: IgG cut-off 0.05; IgM cut-off 0.2. An 80% plaque-reduction neutralization test (PRNT_80_) was performed by using the Hypr 1953 strain to determine the titer of TBEV neutralizing antibodies. Rising TBEV IgG optical density values were detected in a single assay between the earliest and the latest serum specimen (Table). High titers of TBEV neutralizing antibodies were detected on days 11 and 24 after clinical onset of disease (Table). A nested reverse transcription–PCR (*10*) for TBEV in early serum and CSF (days 3 and 4) showed negative results. No TBEV could be isolated by culture on Vero E6 cells.

**Table:** TBEV diagnosis results*†

Samples	Days after clinical onset	ELISA TBEV IgM (OD value)	ELISA TBEV IgG (OD value)	PRNT_80_ TBEV antibody titers	TBEV RT-PCR (*10*)	Virus culture
CSF	3	**1.15**	ND	ND	Negative	ND
CSF	4	ND	ND	ND	Negative	Negative
Serum	4	**1.07**	**0.07**	ND	Negative	Negative
Serum	11	**1.58**	**0.4**	**640**	ND	ND
Serum	24	**1.65**	**1.82**	**640**	ND	ND

*TBEV, tickborne encephalitis virus; IgM, immunoglobulin M; OD, optical density; PRNT80: plaque-reduction neutralization test (80%); RT-PCR, reverse transcription–PCR; CSF, cerebrospinal fluid; ND, not done. **Boldface type** indicates positive values. †IgM capture and IgG indirect in-house assay were performed to detect IgM and IgG TBEV antibodies. The cut-offs were as follows: IgG cut-off 0.05; IgM cut-off 0.2. A PRNT was performed using the Hypr TBEV strain to determine the titer of TBEV-neutralizing antibodies.

## Conclusions

We describe, to our knowledge, the first case of tickborne encephalitis in southwestern France. High levels of TBEV IgM were observed in serum and CSF samples, as is usually observed in TBE neurologic cases (*9*). In addition, the high titers of TBEV-specific antibodies, determined by PRNT, reinforces the conclusion that TBEV is the probable cause of the encephalitis-like condition of the patient. However, no virus was isolated and no genome detected. Early CSF and serum specimens had not been immediately tested for TBEV, and the successive freezing and thawing may explain the negative results. Another explanation could be that the first phase of the disease was not recognized by the patient and that the viremic phase was already finished when the patient was hospitalized, as suggested by the presence of high titers of TBEV-neutralizing antibodies on day 8 after admission. This explanation is compatible with the chronic exposure of the farmer to tick bites. As a differential diagnosis, infection by *Borrelia garinii*, which is also transmitted by *I. ricinus*, could be excluded because no specific IgM and IgG were detectable by serologic testing and no dermatologic signs characteristic of Lyme disease were visible. However, 2 other tickborne viruses, genetically related to TBEV, could not be formally excluded: Louping ill virus and Spanish sheep encephalitis virus.

To date, in France most TBE cases have been reported from Alsace-Lorraine. However, in 2003, 3 cases were reported from the French Alpen region (*5*), likely linked to goat cheese consumption. These new cases raised the question of the extension of TBEV that is endemic in France, as has been observed in Germany and Switzerland. Since 2003, however, patients from this region with meningitis and encephalitis have been more systematically screened for TBEV, and no other case has been identified (I. Schuffenecker, unpub. data).

The discovery of the first TBE case in southwestern France raises the question of the mode of emergence of the virus in this region. The viral cycle involves mainly rodents or deer and ticks with humans as accidental hosts. Domestic ruminants act more as tick transporters than as a reservoir. One hypothesis to the emergence could be the introduction of infected ticks through animal transportation or bird migration. A field survey in the close vicinity of the farmer’s house and fields could yield valuable results. Collecting ticks and rodents could provide the opportunity to identify the circulating strains (as recently was done in Finland and Estonia) (*11*). Also, a seroprevalence study on domestic animals could provide information on the level of circulation of flaviviruses.

Finally, TBEV should be more systematically screened for in patients with encephalitis and meningitis in the absence of any other etiologic diagnosis. Because this finding has implications for expanding vaccine coverage to forestry and agriculture workers, additional epidemiologic data about TBEV circulation in southwestern France should be obtained.
